# Emergency care in post-conflict settings: a systematic literature review

**DOI:** 10.1186/s12873-023-00775-0

**Published:** 2023-04-01

**Authors:** Kalin Werner, Mohini Kak, Christopher H. Herbst, Tracy Kuo Lin

**Affiliations:** 1 Department of Social and Behavioral Sciences, Institute for Health & Aging, University of California, San Francisco, CA San Francisco, USA; 2 Division of Emergency Medicine, University of Cape Town, Cape Town, South Africa; 3 Health, Nutrition and Population Global Practice, The World Bank, Washington, DC USA

**Keywords:** *Post-conflict settings*, *Emergency care*, *Systematic review*

## Abstract

**Background:**

Emergency care systems (ECS) organize and provide access to life-saving care both during transport and at health facilities. Not enough is known about ECS in uncertain contexts such as post-conflict settings. This review aims to systematically identify and summarize the published evidence on the delivery of emergency care in post-conflict settings and to guide health sector planning.

**Methods:**

We searched five databases (PubMed MEDLINE, Web of Science, Embase, Scopus, and Cochrane) in September 2021 to identify relevant articles on ECS in post-conflict settings. Included studies (1) described a context that is post-conflict, conflict-affected, or was impacted by war or crisis; (2) examined the delivery of an emergency care system function; (3) were available in English, Spanish, or French; and (4) were published between 1 and 2000 and 9 September 2021. Data were extracted and mapped using the essential system functions identified in the World Health Organization (WHO) ECS Framework to capture findings on essential emergency care functions at the scene of injury or illness, during transport, and through to the emergency unit and early inpatient care.

**Results:**

We identified studies that describe the unique burden of disease and challenges in delivering to the populations in these states, pointing to particular gaps in prehospital care delivery (both during scene response and during transport). Common barriers include poor infrastructure, lingering social distrust, scarce formal emergency care training, and lack of resources and supplies.

**Conclusion:**

To our knowledge, this is the first study to systematically identify the evidence on ECS in fragile and conflict-affected settings. Aligning ECS with existing global health priorities would ensure access to these critical life-saving interventions, yet there is concern over the lack of investments in frontline emergency care. An understanding of the state of ECS in post-conflict settings is emerging, although current evidence related to best practices and interventions is extremely limited. Careful attention should be paid to addressing the common barriers and context-relevant priorities in ECS, such as strengthening prehospital care delivery, triage, and referral systems and training the health workforce in emergency care principles.

**Supplementary Information:**

The online version contains supplementary material available at 10.1186/s12873-023-00775-0.

## Background

Emergency conditions include a wide range of acute conditions, injuries, communicable and noncommunicable diseases, and complications of pregnancy. Together these conditions contribute substantially to the global burden of disease and represent a key cause of disability and premature death worldwide [1]. Emergency care systems (ECS) organize and provide access to life-saving care by delivering essential health system functions to patients suffering from injury or illness, both during transport and at hospital facilities.

The World Health Organization (WHO) ECS Framework designates essential emergency care functions in to three key categories: (1) at the scene of injury and illness, (2) during transport to an emergency unit (EU), and (3) early inpatient care [2]. Outlining the essential functions of a responsive ECS allows for the characterization of system capacity by mapping the key human resources, equipment, and technologies needed to execute them. In countries where these services are available, ECS often serve as the first point of contact for patients within the health care system by providing diagnosis and the stabilization of acutely ill patients at a critical point of the care continuum. However, in the African region alone it is estimated that only 49% of countries have formal ECS [3].

Early studies indicate a 25% decreased risk in dying from trauma in settings where pre-hospital trauma systems exist [4]. In Northern Iraq and Cambodia low-cost trauma systems were found to reduce trauma mortality from 23.9% to 8.8% [5]. Associated benefits were also significant, including a decreased time from injury to first medical help dropped from 2.4 to 0.6 h [6]. A growing body of literature has started to identify priorities for strengthening emergency care globally using locally appropriate and resource-relevant interventions [7–9]. However, a quarter of the global population live in fragile or conflict affected situations, which presents distinct care delivery challenges and the evidence available to guide decision-makers on context-specific issues of these settings are extremely limited [10].

In post-conflict settings, where the health infrastructure is often undermined, access to health care may be both limited and precarious. For example, emergency care is particularly vulnerable to interruptions in transportation, communication and referral systems [11]. Damaged roads—common in post-conflict settings—limit the ability to dispatch ambulance services, as well as their functionality. Furthermore, weakened health infrastructure reduces the capacity of facilities to deliver and coordinate urgent care. Broken communication infrastructure interrupts system activation and dispatch capacity as well as coordination between emergency crews and complementary services. The lack of adequate infrastructure may lead to primary care facilities being used for medical emergencies despite their lack of capacity to provide such services. The disruption of these key health care services is likely to lead to elevated rates of preventable morbidity and mortality among already- at-risk populations. A better understanding of how these unique challenges interact with emergency care services is a critical first step to expanding access to care and meeting population health needs.

This review aims to systematically identify and summarize the published evidence on the delivery of emergency care in post-conflict settings. Using the essential system functions identified in the WHO ECS Framework, we classify gaps in care delivery and highlight context-relevant priorities, such as prehospital infrastructure and triage and referral systems. The findings will provide policy makers with comprehensive evidence regarding the design of ECS in post-conflict settings and will guide health sector planning.

## Methods

### Study design

A systematic literature review was performed to identify relevant articles on ECS in post-conflict settings. We searched the PubMed MEDLINE, Web of Science, Embase, Scopus, and Cochrane databases in September 2021. The search strategy used context-specific keywords such as “post-conflict settings” in combination with topic-related keywords associated with the delivery of emergency care across functions of scene care, transport care and facility care. Google Scholar was used to search for additional grey literature from the same period. The full search strategy combining relevant keywords using Boolean operators can be found in the table in Supplementary 1.

### Eligibility criteria

Studies were eligible for inclusion if they (1) described a context that is post-conflict, conflict-affected, or was impacted by war or crisis; (2) examined the delivery of an emergency care system function; (3) were available in English, Spanish, or French; and (4) were published between 1 January 2000 and 9 September 2021. Conference abstracts, posters, and protocols were excluded from the review. We followed close definitions of key terms in determining the eligibility of studies (Table 1). However, as the transition between war and peace can be difficult to pinpoint and there is no formal defined time frame which determines post-conflict, we depended on explicit verbal identifiers from study authors to include or exclude papers on this criteria. For example, studies were excluded from the review if the study author(s) did not explicitly include details of conflict, crisis, or war as a feature of the context of the study; such studies were excluded even when the reviewers were aware that conflict had existed in the area. Similarly, “emergency care” was defined by the time-sensitivity of health care services for acute illness or injury [12]. Papers that described disaster medicine or the provision of medical care only during times of emergency or disaster were excluded. As a result, several robust studies that described care delivered during combat or war by American military health services, who play a distinct role in numerous settings of continued conflict, such as Afghanistan, were excluded. Lastly, surgical care was included in our review only if the paper indicated the service was delivered as part of acute or time-sensitive care or was linked to an EU.

**Table 1:** Key definitions used in the review

Term	Definition
Emergency care	*The delivery of health services for conditions that require rapid intervention to avert death or disability, or for which delays of hours can worsen prognosis or render care less effective* [7]
Post-conflict	*A transitional period, characterized by destabilization, where past war or conflict exists on one end and a future period of peace on the other, often most associated with a period of rebuilding and reconstruction*

Microsoft Excel version 16.51 (Microsoft Corporation, Redmond, Washington USA) was used to remove duplicates and for screening and data extraction [13]. Following the Preferred Reporting Items for Systematic reviews and Meta-Analyses (PRISMA) guidelines, two reviewers independently assessed studies for eligibility first by title and abstract, removing those that did not meet the criteria [14]. Full texts of the remaining articles were then retrieved and screened again using the inclusion criteria. Reviewers conducted a risk bias assessment using the Mixed Methods Appraisal Tool (MMAT) to better understand the quality of studies included in our review. However, because of the limited number of studies identified, we did not exclude any papers based on quality [15]. Reviewers checked all within-publication references to identify additional sources. Because this was a desk-based review, no ethical approval was sought.

### Data extraction and analysis

Data elements were abstracted from each included paper by one reviewer and compiled using an extraction matrix. The matrix and variables were discussed between two reviewers for final consensus. The following details were extracted from each included study: country of data origin, study design, authors’ relevant findings, and findings related to each area of the WHO ECS Framework (scene, transport, or facility). In addition, using details and phrases reported in the included papers, authors categorized the type of conflict experienced into one of six categories; protracted conflict, acute ethnic conflict, genocide, acute war, civil war and acute civil unrest. Studies which identified continual, re-emerging or long-term conflict were labeled as protracted, while all other studies were considered acute.

Using the extraction matrix, data were summarized descriptively by country of origin, type of conflict experienced, and study design. Full details extracted from included studies can be found in Table 2. The remaining data were then qualitatively synthesized, with consensus on key themes reached via discussion between the two reviewers. Findings were further extracted and mapped using the WHO ECS Framework to classify gaps in care delivery and highlight context- relevant priorities for essential emergency care functions at the scene of injury or illness, during transport, and through to the EU and early inpatient care.

**Table 2:** Included studies (*n *= 26)

Title	Author(s) (year)	Country origin of data	Type of conflict experienced	Study design	Authors’ relevant findings
Rebuilding the health care system in Afghanistan: An overview of primary care and emergency services	Acerra et al. (2009) [16]	Afghanistan	Protracted conflict	Qualitative	While EDs do exist at some district hospitals and community health centers throughout the country, they are staffed by general practitioners with little or no emergency care training. Local emergency department administrators are not trained to manage EDs. This can be a problem for improving day-to-day operations of the departments
Can patient flow be effectively controlled?	Adini et al. (2011) [17]	Israel	Protracted conflict	Qualitative	It is possible to direct the flow of patients to EDs and rationalize the use of resources, making it possible for patients to be admitted to EDs best able to care for them
Why women die after reaching the hospital: A qualitative critical incident analysis of the ‘third delay' in postconflict northern Uganda	Alobo et al. (2021) [18]	Uganda	Acute war	Interviews	Five reasons were identified for delays: shortage of medicines and supplies, lack of blood and functionality of operating theatres, gaps in staff coverage, gaps in staff skills, and delays in the interfacility referral system. Shortage of medicines and supplies was central in most of the pathways, characterised by three patterns: delay to treat, back-and- forth movements to buy medicines or supplies, and multiple referrals across facilities
Barriers in the delivery of emergency obstetric and neonatal care in post-conflict Africa: Qualitative case studies of Burundi and Northern Uganda	Chi et al. (2015) [11]	Burundi, Uganda	Genocide	Interviews and focus group	The barriers in the delivery of quality EmONC services were categorised into two major themes; human resources-related challenges, and systemic and institutional failures. To improve the situation across the sites, efforts are ongoing to improve the training and recruitment of more staff; harmonise and strengthen the curriculum and training; increase the number of EmONC facilities; and improve staff supervision, monitoring and support
Enhancing governance and health system accountability for people centered healthcare: An exploratory study of community scorecards in Afghanistan	Edward et al. (2015) [19]	Afghanistan	Protracted conflict	Survey	Community score cards are a promising tool for enhancing social accountability for patient-centered care. However, the process requires skilled facilitators to effectively engage communities and healthcare providers and adaptation to specific healthcare contexts
Post-war Kosovo: Part 2 Assessment of emergency medicine leadership development strategy	Eliades et al. (2001) [20]	Kosovo	Acute ethnic civil war	Interviews and focus group	A multi-modal assessment of health systems can provide important information about the need for emergency health system improvements in Kosovo. This methodology may serve as a model for future, system-wide assessments in post-conflict health system reconstruction
Patterns and determinants of pathways to reach comprehensive emergency obstetric and neonatal care (CEmONC) in South Sudan: Qualitative diagrammatic pathway analysis	Elmusharaf et al. (2017) [21]	South Sudan	Protracted conflict	Interviews	Outcomes are better where there is no facility available than when the woman accesses a non-functioning facility, and the absence of a healthcare provider is better than the presence of a noncompetent provider. Visiting non-functioning or partially functioning healthcare facilities on the way to competent providers places the woman at greater risk of dying. Non-functioning facilities and non-competent providers are likely to contribute to the deaths of women
Development of a community-based maternal, newborn and child emergency training package in South Sudan	Fehling et al. (2013) [22]	South Sudan	Protracted conflict	Interviews and focus group)	Significant consensus emerged regarding the need for greater capacity among previously untrained frontline health workers
Mental health status among ethnic Albanians seeking medical care in an emergency department two years after the war in Kosovo: a pilot project	Fernandez et al. (2004) [23]	Serbia and Kosovo	Acute ethnic civil war	Survey	Mental health problems among ED patients in Kosovo, particularly among specific vulnerable populations, are a significant public health concern 2 years after the conflict
Perception of effective access to health services in Territorial Spaces for Training and Reincorporation, one year after the peace accords in Colombia: A cross-sectional study	Fernández-Niño et al. (2020) [24]	Colombia	Protracted conflict	Cross-sectional survey	While residents of Territorial Spaces for Training and Reintegration-Reincorporation regions have a favorable perception of their access to health services, they need to be made aware of extramural and public health activities
Trauma care systems in South Africa	Goosen et al. (2003) [25]	South Africa	Acute civil unrest	Literature review	Gross inequities exist in the provision of trauma care. Access to pre-hospital care and overloading of tertiary facilities are the major inefficiencies to be addressed
A model for emergency medicine education in post-conflict Liberia	Hexom et al. (2012) [26]	Liberia	Civil war	Case report	The use of a global consortium can successfully augment and support academic teaching in emergency medicine in Liberia
Development of emergency medicine in Rwanda	Kabeza et al. (2013) [27]	Rwanda	Genocide	N/A	A program can be designed to provide a sustainable source of locally trained physicians that will work with the countries newly launched EMS system to provide emergency and acute care for a population with great need
Availability of essential health services in post-conflict Liberia	Kruk et al. (2010) [28]	Liberia	Civil war	Survey	One-quarter (26.8%) of the respondents could access basic EmOC. Of the 36 county facilities in Nimba, none (0.0%) provided EmOC. Of the three health centres one (33.3%) provided EmOC. Of the four hospitals all four (100.0%) provided EmOC
Post-war Kosovo: Part 3 Development and rehabilitation of emergency services	Lis et al. (2001) [29]	Kosovo	Acute ethnic conflict	Mixed methods	The majority of emergency patients transported themselves to the hospital. Currently, there only are a few trained, prehospital providers in Kosovo, and almost no airway/cardiac equipment is available on any of the ambulance. There is no centralized emergency department and patients left to self triage. There was no formal triage area, and no materials or equipment was designated for the care and stabilization of ill patients
Punishment attacks in post-ceasefire Northern Ireland: An emergency department perspective	McGarry et al. (2017) [30]	Northern Ireland	Acute ethno-nationalist conflict	Retrospective chart analysis	Punishment attacks continue at a significant rate within the province and there is a return to shooting as the primary means of punishment attack, with an increase in total caseload from 1994. Cost remains a substantial drain on department resources
A mixed methods evaluation of Advanced Life Support in Obstetrics (ALSO) and Basic Life Support in Obstetrics (BLSO) in a resource-limited setting on the Thailand-Myanmar border	McGready et al. (2021) [31]	Thailand, Myanmar	Protracted conflict	Mixed methods	ALSO and BLSO are sustainable, beneficial, EmOC trainings for adult education in protracted, post-conflict, resource-limited settings
Integrating quantitative and qualitative methodologies for the assessment of health care systems: Emergency medicine in post-conflict Serbia	Nelson et al. (2005) [32]	Serbia	Acute ethnic civil war	Mixed methods	Demographic data indicate a loosely ordered network of part-time emergency departments supported by 24-h pre-hospital services and an academic emergency center. Focus groups and questionnaires reveal significant impediments to delivery of care and suggest development priorities
War-related psychological sequelae among emergency department patients in the former Republic of Yugoslavia	Nelson et al. (2004) [33]	Yugoslavia	Acute ethnic conflict	Cross-sectional survey	Three years post-war, symptoms of PTSD and major depression in Serbia remained a significant public health concern, particularly among refugees, those suffering subsequent economic instability, and persons living in rural, remote areas
Post-war development of emergency medicine in Kosovo	O’Hanlon and Lerner (2007) [34]	Kosovo	Acute ethnic conflict	Interviews	Most respondents believed that emergency medicine as a specialised field was a post-war development
First Aid and Voluntarism in England, 1945–85	Ramsden and Cresswell (2019) [35]	England	Acute war	Literature review	Voluntary ambulance services were able to offer solutions to evolving needs and desires such as the increased demand for first-aid training in the workplace, the need for first-aid cover in a more leisure oriented society
Prehospital injury severity of children evacuated by helicopters from combat zones: A retrospective report	Samuel et al. (2013) [36]	Israel	Protracted conflict	Retrospective comparative	In the prehospital setting, children evacuated from combat zones were more severely injured than children who were transported from the scene during peacetime
Providing emergency care and assessing a patient triage system in a referral hospital in Somaliland: A cross-sectional study	Sunyoto et al. (2014) [37]	Somaliland	Protracted civil war	Cross-sectional	The high proportion of late presenters to the ED suggests considerable barriers to care and lack of awareness amongst patients and the community on the need of early presentation. ED staff was able to use this system accurately and reflecting positively on the training programme provided
A first aid training course for primary health care providers in Nagorno Karabagh: Assessing knowledge retention	Thompson et al. (2012) [38]	Azerbaijan	Acute ethnic conflict	Survey	The trainees assessed the first- aid training course as effective, and the skills covered as important and well utilized. Knowledge retention was modest, but stable. Refresher courses are necessary to reverse the decay of technical knowledge and to ensure proper application in the field
Post-war Kosovo: Part 1. Assessment of prehospital emergency services	Vanier et al. (2001) [39]	Kosovo	Acute ethnic conflict	Interviews	By improving the communications, staffing, equipment, and transport patterns in the system, significant progress can be realized in expanding Kosovo's emergency care capabilities
Existing infrastructure for the delivery of emergency care in post-conflict Rwanda: An initial descriptive study	Wen and Char (2011) [40]	Rwanda	Genocide	Interviews	The three most commonly cited problems facing EM infrastructure in Rwanda were lack of resources (94% of respondents), need for specialised EM training (89%), and absence of prehospital care (74%). ongoing challenges, specifically with continuing lack of resources, need for specialised EM training, and deficiency of prehospital care, along with new questions about sources of funding and implications for the healthcare workforce

## Results

**Fig. 1:**
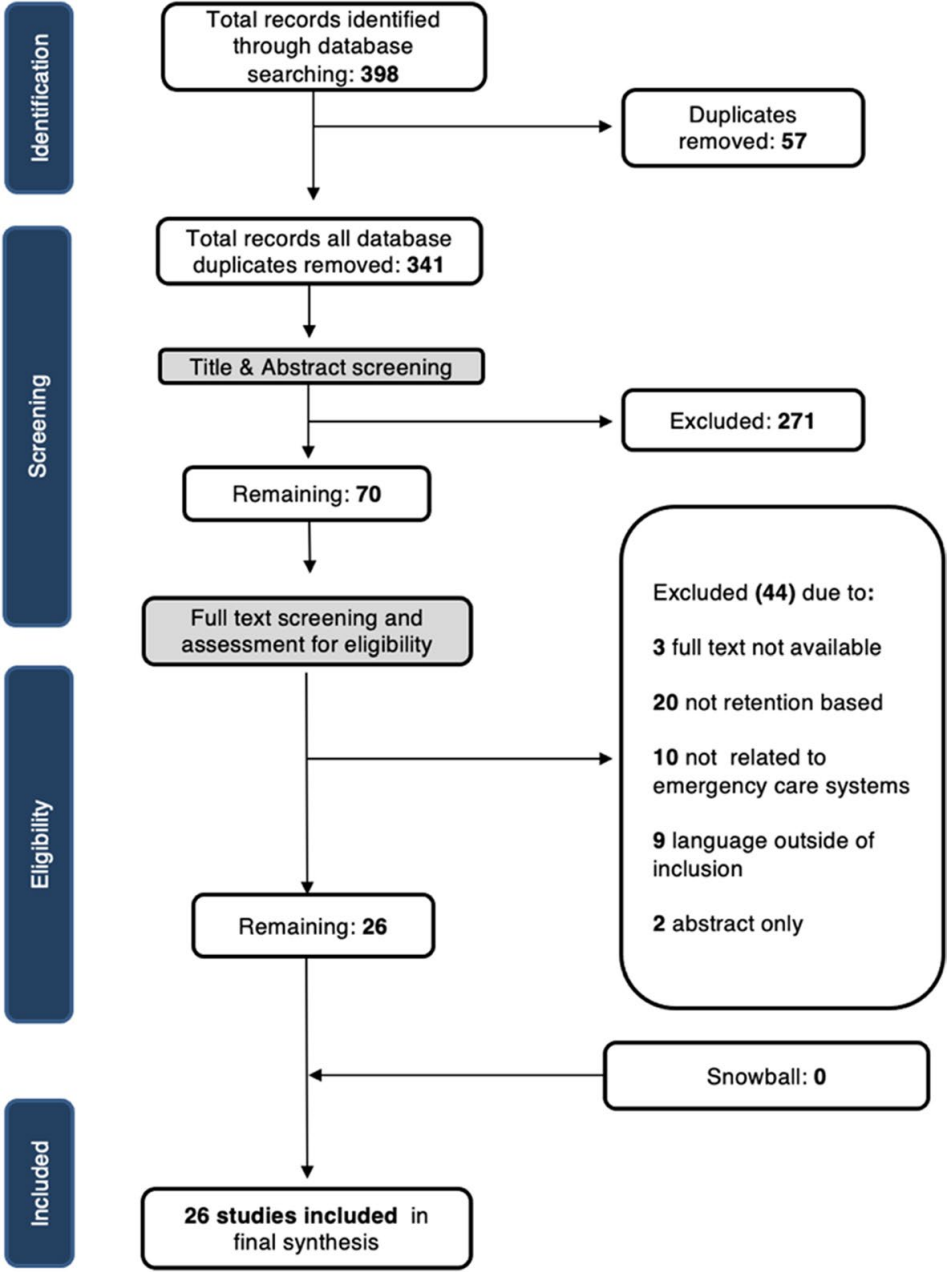
PRISMA diagram of included studies

Our search identified 398 studies, after which 57 duplicates were removed (Fig. 1). Two reviewers assessed the titles and abstracts of the remaining 341 papers based on the inclusion criteria, resulting in the exclusion of another 271 papers. Seventy potentially eligible papers were included in our full-text review. Of these, 20 papers were not related to ECS, 10 were not conducted in post-conflict settings, 9 were published in a language outside of our inclusion list, 3 were not available, and 2 were available only as conference abstracts. In addition, reviewers searched the references lists of all studies included during full-text review. This process did not identify any additional studies that were added to our review. Twenty-six papers were included in our final review.

### Study characteristics

A wide range of countries (*n* = 18) were represented in our review. The majority of papers analyzed delivery of emergency care in either the Sub-Saharan Africa region (*n* = 11) or Europe and Central Asia region (*n* = 10). Kosovo and/or Serbia (*n* = 6) were the most well represented, followed by Afghanistan (*n* = 2), Israel (*n* = 2), Rwanda (*n* = 2), South Sudan (*n* = 2), and Uganda (*n* = 2). The most common type of conflict was protracted conflict (n = 9), followed by acute ethnic conflict (*n* = 9). Three studies were from contexts of genocide, two from instances of acute war, two from civil war, and one from acute civil unrest.

Most of the studies in our review were descriptive and qualitative (*n* = 17), with 8 using interview or focus group methodologies and 7 using cross-sectional survey methodologies. Three papers used mixed methods approaches [26, 33, 39], two conducted retrospective chart reviews [19, 41], and 1 is presented as a case report [16].

Results of the risk of bias scoring exercise can be found in Table 3. Over half (*n* = 15) of the studies were considered to have a low risk of bias and high quality (scores of 6 and above), while eight studies were considered to be of poor quality (scores of 4 or 5). Three low-quality studies scored less than half of the total available seven points and would be considered at high risk of bias.

**Table 3:** Risk of bias assessment scores

	**Screening questions**		**Qualitative study questions**					**Quantitative descriptive study questions**	
**Author (Year)**	*Are there clear research questions?*	*Do the collected data allow to address the research questions?*	*Is the qualitative approach appropriate to answer the research question?*	*Are the qualitative data collection methods adequate to address the research question?*	*Are the findings adequately derived from the data?*	*Is the interpretation of results sufficiently substantiated by data?*	*Is there coherence between qualitative data sources, collection, analysis and interpretation?*	*Is the sampling strategy relevant to address the research question?*	*Is the sample representative of the target population?*
Acerra et al. (2009) [16]	N	N	N	N	N	N	N	n.a	n.a
Adini et al. (2011) [17]	Y	Y	n.a	n.a	n.a	n.a	n.a	Cannot tell	Y
Alobo et al. (2021) [18]	Y	Y	Y	Y	Y	Y	Y	n.a	n.a
Chi et al. (2015) [11]	Y	Y	Y	Y	Y	Y	Y	n.a	n.a
Edward et al. (2015) [19]	Y	Y	n.a	n.a	n.a	n.a	n.a	Y	Y
Eliades et al. (2012)	Y	Y	n.a	n.a	n.a	n.a	n.a	Y	Cannot tell
Elmusharaf et al. (2017) [21]	Y	Y	Y	Y	Y	Y	Y	n.a	n.a
Fehling et al. (2013) [22]	Y	Y	Y	Y	Y	Y	Y	n.a	n.a
Fernandez et al. (2004) [23]	Y	Y	n.a	n.a	n.a	n.a	n.a	Y	Y
Fernández-Niño et al. (2020) [24]	Y	Y	n.a	n.a	n.a	n.a	n.a	Y	Y
Goosen et al. (2003) [25]	Y	Y	Y	N	Y	Y	Y	n.a	n.a
Hexom et al. (2012) [26]	Y	Y	n.a	n.a	n.a	n.a	n.a	n.a	n.a
Kabeza et al. (2013) [27]	Y	Y	n.a	n.a	n.a	n.a	n.a	n.a	n.a
Kruk et al. (2010) [28]	Y	Y	n.a	n.a	n.a	n.a	n.a	Y	Y
Lis et al. (2012)	Y	Y	n.a	n.a	n.a	n.a	n.a	n.a	n.a
McGarry et al. (2017) [30]	Y	Y	n.a	n.a	n.a	n.a	n.a	Y	Cannot tell
McGready et al. (2021) [31]	Y	Y	n.a	n.a	n.a	n.a	n.a	n.a	n.a
Nelson et al. (2005) [32]	Y	Y	n.a	n.a	n.a	n.a	n.a	n.a	n.a
Nelson et al. (2004) [33]	Y	Y	n.a	n.a	n.a	n.a	n.a	Y	N
O'Hanlon and Lerner (2011)	Y	Y	n.a	n.a	n.a	n.a	n.a	n.a	n.a
Ramsden and Cresswell (2019) [34]	Y	Y	n.a	n.a	n.a	n.a	n.a	n.a	n.a
Samuel et al. (2013) [36]	Y	Y	n.a	n.a	n.a	n.a	n.a	N	Cannot tell
Sunyoto et al. (2014) [37]	Y	Y	n.a	n.a	n.a	n.a	n.a	Y	Cannot tell
Thompson et al. (2012) [38]	Y	Y	n.a	n.a	n.a	n.a	n.a	Y	N
Vanier et al. (2012)	Y	Cannot tell	n.a	n.a	n.a	n.a	n.a	n.a	n.a
Wen and Char (2011) [40]	Y	Y	n.a	n.a	n.a	n.a	n.a	n.a	n.a

	**Quantitative descriptive study questions**			**Mixed methods study questions**					
**Author (Year)**	*Are the measurements appropriate?*	*Is the risk of nonresponse bias low?*	*Is the statistical analysis appropriate to answer the research question?*	*Is there an adequate rationale for using a mixed methods design to address the research question?*	*Are the different components of the study effectively integrated to answer the research question?*	*Are the outputs of the integration of qualitative and quantitative components adequately interpreted?*	*Are divergences and inconsistencies between quantitative and qualitative results adequately addressed?*	*Do the different components of the study adhere to the quality criteria of each tradition of the methods involved?*	**TOTAL SCORE (OUT OF 7)**
Acerra et al. (2009) [16]	n.a	n.a	n.a	n.a	n.a	n.a	n.a	n.a	0
Adini et al. (2011) [17]	Y	Cannot tell	Y	n.a	n.a	n.a	n.a	n.a	5
Alobo et al. (2021) [18]	n.a	n.a	n.a	n.a	n.a	n.a	n.a	n.a	7
Chi et al. (2015) [11]	n.a	n.a	n.a	n.a	n.a	n.a	n.a	n.a	7
Edward et al. (2015) [19]	Y	Cannot tell	N	n.a	n.a	n.a	n.a	n.a	5
Eliades et al. (2012)	Y	Cannot tell	N	n.a	n.a	n.a	n.a	n.a	4
Elmusharaf et al. (2017) [21]	n.a	n.a	n.a	n.a	n.a	n.a	n.a	n.a	7
Fehling et al. (2013) [22]	n.a	n.a	n.a	n.a	n.a	n.a	n.a	n.a	7
Fernandez et al. (2004) [23]	Y	Cannot tell	Y	n.a	n.a	n.a	n.a	n.a	6
Fernández-Niño et al. (2020) [24]	Y	Y	Y	n.a	n.a	n.a	n.a	n.a	7
Goosen et al. (2003) [25]	n.a	n.a	n.a	n.a	n.a	n.a	n.a	n.a	6
Hexom et al. (2012) [26]	n.a	n.a	n.a	Y	Y	N	N	N	4
Kabeza et al. (2013) [27]	n.a	n.a	n.a	N	Y	N	N	N	3
Kruk et al. (2010) [28]	Y	Y	Y	n.a	n.a	n.a	n.a	n.a	7
Lis et al. (2012)	n.a	n.a	n.a	Y	Y	Y	Cannot tell	Y	6
McGarry et al. (2017) [30]	Y	Cannot tell	Y	n.a	n.a	n.a	n.a	n.a	5
McGready et al. (2021) [31]	n.a	n.a	n.a	Y	Y	Y	Y	Y	7
Nelson et al. (2005) [32]	n.a	n.a	n.a	Y	Y	Y	Y	Y	7
Nelson et al. (2004) [33]	Y	Y	Y	n.a	n.a	n.a	n.a	n.a	6
O'Hanlon and Lerner (2011)	n.a	n.a	n.a	Y	Y	Y	Y	Y	7
Ramsden and Cresswell (2019) [35]	n.a	n.a	n.a	N	N	Y	Cannot tell	Y	4
Samuel et al. (2013) [36]	Y	Y	Y	n.a	n.a	n.a	n.a	n.a	5
Sunyoto et al. (2014) [37]	Y	Y	Y	n.a	n.a	n.a	n.a	n.a	6
Thompson et al. (2012) [38]	Y	Cannot tell	Y	n.a	n.a	n.a	n.a	n.a	5
Vanier et al. (2012)	n.a	n.a	n.a	N	Y	Y	N	N	3
Wen and Char (2011) [40]	n.a	n.a	n.a	Y	Y	Y	Y	Y	7

*na* not applicable

Four studies reported on the unique burden of disease in EUs in post-conflict settings as a result of years of trauma and conflict. In one instance, punishment attacks, or paramilitary-style shootings, beatings and injuries born from the ethno-nationalist conflict in Northern Ireland, remain a common hospital presentation, and the costs of treating these attacks remain a substantial drain on EU resources throughout the post-conflict period [19]. Furthermore, populations in post-conflict settings may suffer from war-related psychological sequelae and carry an elevated mental health burden of disease [42]. War-related dysfunction of the health system impacts care for patients presenting in post-conflict contexts. For example, in Kosovo, survey participants identified the leading emergencies to be trauma, cardiac disease, ingestions, and suicidality [36, 43]. These sequelae are further impacted by a broad lack of system health infrastructure in post-conflict settings, which results in patients with primary health care needs seeking care in EUs rather than in primary care clinics. For example, one study in Kosovo assessed the mental health of ethnic Albanians presenting for non-acute EU care and found that there are significant public health concerns in mental health [30]. Another study found that nearly 50% of EU patients responding to a survey in two Serbian communities had symptoms consistent with depression, and 13% of their symptoms were consistent with post-traumatic stress disorder [23].

The patterns of burden of disease were also reported to shift as states transition from periods of conflict to a post-conflict setting. For example, in South Africa, it was noted that the transition from conflict to post-conflict democracy accompanied a decline in stab wounds. The same study also indicated that the transition is associated with an increase in gunshot wounds [29].

**Fig. 2:**
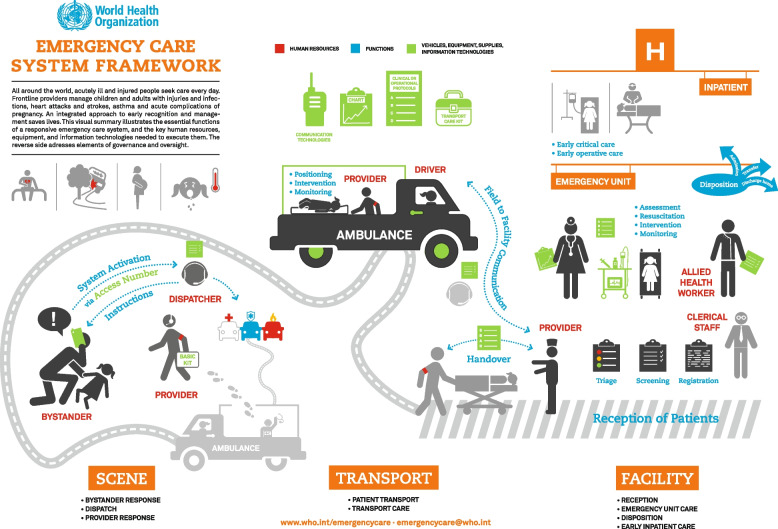
WHO ECS Framework

We use the WHO ECS Framework to further organize the findings from the literature and bring together information for a wide variety of contexts and time periods in a simple and straightforward manner. (Fig. 2) [2]. This framework characterizes system capacity by establishing essential emergency care functions in three key areas: at the scene of injury and illness, during transport to the EU, and early inpatient care. Detail of the findings of the literature each of these areas is summarized in Table 4 with further detail provided below.

**Table 4:** Findings related to scene care

Bystander response	Dispatch	Provider response
Volunteers deliver emergency first-aid assistance during bombing raids in England [39]	Unreliable telephone system prevents patients from activating ambulance response in Kosovo [40]	No formal prehospital triage system in Rwanda [28]

### Scene care

Scene care is delivered in the community by bystanders, dispatchers, and providers. Bystanders activate the response system by calling an access number. Dispatchers apply operational protocols and relay instructions back to bystanders on the scene and to providers who report to scene with a basic emergency kit to provide care. Details of the scene elements—such as bystander response, dispatch, and provider response—were reported in only three articles (Table 4). Dispatch capacities were strained in all cases. One study in Rwanda found that prehospital care was almost entirely missing as was any formal prehospital triage or dispatch, which limits any ability to divert patients to appropriate care [44]. Another study, in Kosovo, found that although an emergency telephone system did exist in the country, patients were rarely able to activate provider response because the telephone system for the country was unreliable [45]. Volunteer emergency first-aid assistants were documented in another study to commonly occur in Britain during World War II. The voluntaristic commitment to the communal and national good fostered during the war did not fade, even as the country transitioned into a post-conflict period and the state absorbed the responsibility for individuals’ welfare [20].

### Transport

Transport includes both efforts to transport patients to facilities and the care delivered during the journey. Key human resources include providers and ambulance drivers. Providers, using clinical protocols, initiate care by conducting critical interventions as well as positioning and monitoring the patient while providing key field-to-facility communication. Our review identified five studies that reported results specific to patient transport or transport care (Table 5) [11, 16, 25, 27, 30].

**Table 5:** Findings related to transport care

Patient transport	Transport care
Majority of patients self-transport in Kosovo [20]	Few trained prehospital providers and almost no airway/cardiac equipment are available on ambulances in Kosovo [20]
No national or provincial emergency reporting system in Rwanda, and a few ambulances but not systematic use to respond to emergencies or transfer patients to higher levels of care [28]	Few ambulances have adequate space for providing treatment to patients, while the rest of the transport vehicles are compact automobiles in Kosovo [40]
47.6% of patients with injuries arrive at hospital by private vehicle in South Africa [18]	Volunteer ambulance services are able to offer first-aid assistance in England [39]

Nearly all studies reported a lack of formal ambulances with adequate equipment. For example, in South Africa, almost half of all injuries arrive at the hospital by private vehicle rather than by ambulance [29]. Similar findings were exhibited in Kosovo, where the majority of emergency patients were found to transport themselves to the hospital because there were few trained prehospital providers [39]. On the limited number of ambulances, little or no airway or cardiac equipment was available on board [39]. In Rwanda, very limited prehospital infrastructure was found; there were no national or provincial emergency reporting systems [44]. Few ambulances with adequate space were found to be in service in the ECS in Kosovo, although numerous compact automobiles were used to transport patients [45]. Studies in Kosovo mentioned the use of personal vehicles, taxis, or commercial vehicles without critical emergency care equipment [11, 25], highlighting the fact that, although patients are still arriving at facilities, there is a lack of protocol-based response and a lack of transport systems equipped to respond to emergencies. On the other hand, in one study in Israel, airborne rescue and evacuation units were integrated into national health care systems during times of both peace and conflict, providing critical pediatric trauma care in the country [41]. In England, volunteer ambulance services played a key role in providing first-aid assistance to patients [20].

Scene and transport care both occur prior to arrival at a facility and can both be encompassed in the term “prehospital care.” A majority of the literature identified the lack of prehospital care as a major contributor to the overall inadequate ECS infrastructure [11, 23, 25, 27]. As a result, this area appeared to be the weakest point of the ECS in post-conflict settings.

### Facility

Facilities are the point in the ECS where patients are expected to receive definitive care, which heavily relies on appropriate triage, adequate supplies and staff, and physical infrastructure. Here key human resources include providers, allied health workers, and clerical staff. When arriving at facilities, ambulance providers, following clinical protocols, hand patients over to facility providers, who then conduct triage and patient screening. Clerical staff then complete patient registration. Patients are subsequently managed by allied health workers during their stay in the EU; after this they are either discharged home, transferred to another facility, or admitted to the inpatient ward for early critical or operative care.

Several studies reported results related to the responsibilities of facility care such as reception, EU care, and disease presentation at EUs. None of the included studies had a focused discussion on patient disposition or early inpatient care (Table 6).

**Table 6:** Findings related to facility care

Reception	Emergency unit care	Disease presentation
Local emergency department administrators are not trained to manage EUs in Afghanistan [36]	No centralized EU, patients left to self-triage in Kosovo [39]	Symptoms consistent with PTSD and major depression in nearly half of patients presenting for care [19, 41]
	EDs staffed by general practitioners with little or no emergency care training [36]	
	No specialty societies or post-graduate training programs in EM were identified in Rwanda [44]	
	Need for greater capacity among untrained frontline health care workers [37]	
	Bulk of trauma managed at regional hospital level [29]	

*ED* emergency department, *EU* emergency unit, *PTSD* post-traumatic stress disorder

In Kosovo, limited equipment and inadequate materials were available in hospitals to stabilize and care for patients [39]; in Afghanistan, it was noted that hospitals outside of main city centers struggled to provide anything beyond rudimentary care because of a lack of equipment, electricity, and running water [36]. Similar results were identified in South Africa, where a majority of the trauma was found to be managed in better-equipped regional hospitals rather than rural ones [29]. In Liberia, only one-quarter of respondents could access basic emergency obstetric care (EmOC), and none of the county-level facilities provided these services [40].

Studies also identified a notable lack of triage systems used to assess patient severity and prioritize the delivery of care. These systems are critical to supporting the rational use of limited resources [20, 45]. In some cases, such as Kosovo, no centralized emergency departments were identified; as a result, patients presenting to hospitals with emergencies were left to self-triage. One study in Afghanistan identified that local staff were often not trained to manage EUs, which resulted in inefficient unit operations and practices. However, a study conducted in Israel found that one way to remedy inefficient operations was to direct the flow of patients to EUs—decreasing admissions in a level-I trauma center while raising admissions in two other level-II hospitals [34]. One study validated the use of the South African Triage Score (SATS) system by applying the system in Somaliland, finding that triage systems that are suitable to the context already exist and could be applied to post-conflict settings immediately [28].

In most post-conflict settings where EUs were available, they were staffed by general practitioners with little or no emergency care training [36]. In Azerbaijan, training primary health care providers in first aid was found to improve the skills and knowledge of trainees [18].

## Discussion

To our knowledge, this is the first study to systematically identify the evidence on ECS in fragile and conflict-affected settings. We found studies that describe the unique burden of disease and challenges in delivering to the populations in fragile and conflict-affected states, pointing to particular gaps in prehospital care delivery (both during on-scene response and during transport). Our review further highlights several common barriers limiting the delivery of emergency care in post-conflict settings, including (1) poor infrastructure, (2) lingering social distrust, (3) scarcity of formal emergency care training, and (4) a critical lack of resources and supplies (Table 7).

**Table 7:** Findings related to each area of the WHO ECS framework in post-conflict settings

	**Scene**	**Transport**	**Facility**
Key barriers	Poor infrastructure		Lingering social distrust
			Scarcity of formal emergency care training
		Lack of resources and supplies	
Interventions assessed in our review	NA	Increase community awareness of essential package of care	Deliver emergency care and medical education services by expatriates
			Advanced Life Support in Obstetrics and Basic Life Support in Obstetrics training
		Initiate civilian ambulance programs	Apply existing triage systems to local setting
			Control patient flow to metropolitan emergency units

*NA* not available

A majority of the literature in our review were descriptive in nature, likely indicating the relative novelty of emergency care as a focus and specialty in these settings. The results of this systematic literature review play an important role in understanding the needs across various settings and establishing the foundation of understanding the context of emergency care delivery in post-conflict settings. Limited access to care was commonly reported. In Colombia, although 20.8% of ex-combatant survey respondents reported that they or a member of their household was in need emergency care services due to illness, only 16.4% had sought care [22]. In Somaliland, a high proportion of late presenters to the EU indicated that access to care and awareness of the need to seek timely care may be low among patients [28]. Triage systems that are a critical component of the rational use of limited resources and that ensure that patients are diverted to the appropriate level of care were also severely lacking [20, 45].

### Poor infrastructure

The relationship between conflict and the development of ECS is complicated and likely endogenous. In one case, the growth of emergency care was recognized as a post-conflict development, indicating that, in some contexts that have underdeveloped emergency care, a post-conflict setting could be a uniquely appropriate time to engage with ECS development efforts [27]. Other studies noted the destruction of key infrastructure throughout periods of conflict; events such as bombings were particularly damaging to the delivery of emergency care because of the loss of passable roads and limited communication infrastructure to assist dispatching of emergency care services [6, 25]. Post-conflict settings also appear to face specific challenges in delivery of care because the lingering effects of war such as the loss of usable, functioning equipment in facilities, resulting in facilities failing to be equipped to function [45]. These factors contributed to clear deficiencies in prehospital care across nearly all included studies. For the most part, pathways for emergency care—whether for mothers seeking EmOC or for patients needing general trauma care—were both protracted and precarious [24].

### Lingering social distrust

Even when conflict has come to an end, it may be difficult to repair the distrust between opposing groups. The challenges of social cohesion extend into health service and delivery. One study found that, following the Serbian occupation of Kosovo, opposing ethnic groups avoided seeking care from the historical opposition, preferring instead to seek care from their own ethnic groups. Rather than being treated in Serbian-run government facilities, many Albanians sought care at private clinics that they knew to be run by Albanians [45]. Concerns regarding remaining social tensions and installing equity in health care delivery for marginalized populations was a challenge in various locations [11, 36]. In Afghanistan, enhanced sense of mutual trust between the community and medical providers was achieved through community score cards [16]. A growing body of research points to the role of the health system in strengthening the social contract and reducing inequities in post-conflict settings [35] 

### Scarcity of formal emergency care training

Health care worker training and expertise, particularly regarding issues of formal training in emergency care principles, was another common challenge identified in the literature [20, 28, 32, 36, 46]. This barrier is especially critical, as incompetent providers may lead to a higher mortality and morbidity rate. One study found that outcomes for women who need EmOC are better in the absence of a health care provider than in the presence of a non-competent provider [47].

Efforts to harmonize and strengthen emergency care curriculum and training already appear to be underway. Models for emergency care education, which utilized consortiums of expatriates to teach local health care workers and students, have been reported in Liberia and Rwanda [20, 46]. Educational interventions were found to have long-term effects on wound management principles and blood/fluid precautions. Although the impact of educational training on advanced airway interventions and trauma resuscitation procedures have not been found to be as considerable as the impact of training in wound management principles and blood/fluid precautions [48].

The need for specialized and improved emergency care training across all cadres, not only specialists, was also evident. First-aid training of primary health care providers was found to successfully improve skills and knowledge in emergency care, although knowledge retention refresher courses in the future are recommended to maintain the knowledge gained [25]. On the Thailand-Myanmar border, Advanced Life Support in Obstetrics and Basic Life Support in Obstetrics courses were found to have both self-declared benefits for health care worker knowledge, skills, and teamwork and to decrease the risk of post-partum hemorrhage related to maternal mortality and stillbirth [26].

It is likely that the lack of formal training has negative impacts prior to arrival at facility. Studies in our review did not make specific mention of protocol availability, however, evidence indicates that appropriate medical protocols and checklists improve care process measures and reduce mortality where care is provided by informally trained staff [21, 49].

### Lack of resources and supplies

Finally, resource-related challenges were endemic across the various settings, limiting the capacity to rehabilitate or develop emergency care services. For example, facilities in Afghanistan were found to be inadequately stocked so they could not ensure treatment of patients with emergencies; they were also found to have a dearth of appropriate medications, supplies, and equipment [36] Numerous studies indicated that equipping facilities is a critical next step in improving ECS in a post-conflict setting, reinforcing the need to improve the availability of financial resources to further develop emergency care [27].

Incidentally, one paper juxtaposed the lack of emergency care capacity to the relatively widespread availability of HIV services within the same setting and suggested that this is possibly a result of consistent large donor investments in that specific sector, along with donor-enforced health sector priorities [40]. Unfortunately, global funding for ECS is sparse, especially when contrasted to that for vertical disease programmes, such as HIV/AIDs, TB, or malaria. This is reflective of the wider health financing environment in low-resource health systems where development aid and donor assistance may be responsible for almost a quarter of health spending [50]. This finding points toward the need for stronger advocacy and awareness of the critical role of emergency care, and increased donor funding particularly in settings where health systems may already be fragile and where the populations are disadvantaged and marginalized.

Furthermore, nine of the studies in our review derive from countries experiencing protracted conflict. Although none measure the extent to which this lingering insecurity influences access to care, it is reasonable to believe that post-conflict settings which continue to experience insecurity face our identified barriers to a further extent.

### Limitations

The lack of a standard definition or ability to pinpoint the exact moment that a setting is considered post-conflict or during conflict means that some studies from countries excluded in our review might have been included if a different set of criteria had been applied to the review. To ensure consistency and transparency, we defined the scope of our search by following the strict definition of post-conflict indicated in Table 1.

Similarly, the parameters regarding what falls within and what falls outside of the scope of ECS interventions can be limiting, especially in contexts where there lacks formally organized ECS. For example, studies focusing on the implementation of disaster management elements of disaster medicine, rather than the provision of emergency care services during natural and man-made emergencies, were not included in our review. Although we did our best to include terms such as “trauma,” “acute care,” and “injury” to capture the broadest definition of the term, as well as terms specific to various functions of the ECS, there may be relevant articles that were not caught by our search criteria as a result of the limitations of language to definitively define “emergency care.” For this reason, we further hand-searched references of included articles. Finally, results were derived from diverse environments and periods, ranging from Europe during World War II to recent conflicts in Sub-Saharan Africa, which may limit their comparability.

Lastly, we systematically excluded papers that discussed only combat medicine even though we acknowledge that post-conflict settings frequently vacillate between periods of active conflict and peace, and in these settings, it may be more likely for the lines between combat and emergency medicine to be blurred. This choice may have resulted in the exclusion of some relevant findings to the setting, although the primary aim of this study was to guide rational and sustainable health sector planning that occurs outside of perpetual conflict and combat.

### Lessons for establishing emergency care in post-conflict settings

Very few papers identified in our review sought to assess the effectiveness of approaches or interventions to address these barriers to emergency care (*n* = 3). Although our review identified some notable findings, generalizing any such results should be done with caution because of the significant heterogeneity of post-conflict settings.

In Afghanistan, unrealistic community demands for ambulances and specialists were mitigated by educating the community to increase awareness of their essential package of care entitlements [38]; it was recommended that civilian ambulance programs be used in urban areas to begin establishing ECS [36]. In Israel, where resources in more rural areas—as compared to urban areas—were scarce, redirecting the patient flow to EDs in a metropolitan area made it possible for patients to be admitted to the EDs best able to care for them [46]. Applying existing triage systems, such as the South African Triage Score, was feasible in post-conflict district hospitals in Somaliland [28]; the findings suggest that the triage system may be applicable in other post-conflict settings. Finally, among a variety of efforts to improve training, including the delivery of emergency medical care and medical education services by expatriates, educational seminars were found to have the greatest sustained effect on provider behaviors [18, 39].

Many functions of ECS, such as scene care, are suited to community-based care efforts. Our review identified a modest amount of evidence regarding the appropriateness of training packages for community-based emergency care in maternal and child health, bystander first-aid, and volunteer ambulance support in post-conflict settings [20, 37]. Such results align with the broader literature on the substantial effects of first-aid training and task shifting to laypeople to reduce morbidity and mortality in low-resource settings [51].

Although the descriptive studies identified in our review provide an important starting place, few studies used any form of quantitative means to assess interventions aimed at improving the delivery of emergency care. Thus, quantitative analyses that can provide evidence on the effectiveness of interventions for improving emergency care in these settings are lacking. Future research should endeavor to corroborate the effectiveness of emergency care interventions that have been demonstrated to be effective in low-resource settings, in the context of post-conflict settings. To start, interventions to consider include implementing triage systems [52], training health care providers in basic emergency care skills [41, 53–56], and conducting first-aid responder trainings [31, 51].

## Conclusion

Aside  from managing urgent conditions, ECS deliver competencies that are essential for public health emergencies, conflict settings, and disasters, and these systems can improve access for patients. These systems play a central role in the delivery of a range of essential emergency care services that contribute to the achievement of universal health coverage. Calls to align ECS with existing global health priorities would ensure access to these critical life-saving interventions, which could save more than 400,000 lives each year across the globe [54]. In 2019, World Health Assembly resolution 72.16 noted the capacity of integrated emergency care to save lives and maximize impact across the health system and also raised concerns over the lack of investments in frontline emergency care [53]. There is an emerging understanding of the state of emergency care systems in post-conflict settings; however, the current body of evidence related to best practices and interventions is extremely limited. As states seek to rebuild their health care systems post-conflict, careful attention should be paid so as to address the common barriers and context-relevant priorities in the provision of ECS, such as strengthening prehospital care delivery and training the health workforce in emergency care principles.

## Supplementary Information

**Additional file 1: Supplementary 1. **Search Strategy. 

## Data Availability

All data generated or analyzed during this study are included in this published article and its supplementary information file.

## Abbreviations

ECS: Emergency care systems
ED: Emergency department
EmOC: Emergency obstetric care
EU: Emergency unit
MMAT: Mixed Methods Appraisal Tool
PRISMA: Preferred Reporting Items for Systematic reviews and Meta-Analyses
SATS: South African Triage Score
WHO: World Health Organization
