# Supplementary material for: Emergency care in post-conflict settings: a systematic literature review
Source: BMC Emerg Med. 2023 Apr 1;23:37. doi: 10.1186/s12873-023-00775-0 (PMC10068156; doi:10.1186/s12873-023-00775-0)
Supplement: Supplementary file 1 — Additional file 1: Supplementary 1. Search Strategy. [file 12873_2023_775_MOESM1_ESM.docx]

**Supplementary 1. Search Strategy**

| *Last modified and run 9 September 2021* | |
| --- | --- |
| **DATABASE (n)** | SEARCH STRINGS |
| **PubMed MEDLINE (134)** | 1 (emergency medical service? OR emergency medical technician? OR emergency treatment? OR emergency medicine OR emergency service? OR emergency department? OR emergency room? OR emergency ward? OR emergency unit? OR emergency cent? OR trauma cent? OR emergency health service? OR accident and emergency OR accident & emergency OR a&e OR A&E OR ambulance? OR air ambulance? OR first aid OR prehospital OR pre-hospital OR paramedic? OR out-of-hospital OR out of hospital OR ems OR emt OR emergency medical service? OR emergency technician? OR emergency practitioner OR emergency dispatch? OR first responder OR emergency rescue? OR emergency resus? OR emergency triage OR advanced life support OR emergency care practitioner OR prehospital OR pre-hospital OR emergency medical technicians OR emergency service? OR field triage OR out-of-hospital OR emergency medical service communication system? OR emergency medical dispatch? OR medical dispatch? OR telephone triage OR ambulance dispatch? OR Medical Priority Dispatch System).tw. 2 (post-conflict OR post conflict OR post-conflict setting OR postconflict setting OR post-conflict country OR postconflict country OR post-conflict countries OR post conflict countries OR post-conflict zone OR post conflict zone OR post-conflict region OR post conflict region OR post-war OR postwar OR post-war setting OR postwar setting OR post-war state OR postwar state OR post-war country OR postwar country OR post-war countries OR postwar countries OR post-war zone OR postwar zone OR post-war region OR postwar region) .tw. 3 (health system reconstruction OR health service reconstruction OR fragile setting OR fragile state OR fragile states OR fragile nation OR fragile nations OR fragile country OR fragile countries OR fragile region OR fragile regions OR conflict recovery) .tw. 4 or/2-3  5 1 and 4  6 limit 5 to yr= “2000 -Current" |
| **Embase (77)** | 1 ‘emergency medical service?’ OR ‘emergency medical technician?’ OR ‘emergency treatment?’ OR ‘emergency medicine’ OR ‘ambulance?’ OR ‘air ambulance?’ OR ‘first aid’ OR ‘prehospital’ OR ‘pre-hospital’ OR ‘paramedic?’ OR ‘out-of-hospital’ OR ‘out of hospital’ OR ‘ems’ OR ‘emt’ OR ‘emergency service?’ OR ‘emergency technician?’ OR ‘emergency practitioner?’ OR ‘emergency dispatch?’ OR ‘first responder’ OR ‘public access defibrillation’ OR ‘emergency rescue?’ OR ‘emergency resus?’ OR ‘paramedic?’ OR ‘emergency triage’ OR ‘advanced life support’ OR ‘emergency care practitioner?’ OR ‘extended care practitioner?’ OR ‘HEMS’ OR ‘field triage’ OR ‘emergency service?’ 2 ‘emergency medical service communication system?’ OR ‘emergency medical dispatch?’ OR ‘medical dispatch?’ OR ‘telephone triage’ OR ‘ambulance dispatch’ OR ‘Medical Priority Dispatch System’ OR ‘MPDS’ 3 #1 OR #2  4 (‘post-conflict*’ or ‘combat’) next/2 (‘area?’ or ‘zone?’ or ‘setting?’ or ‘region?’ or ‘military’ or ‘armed’ or ‘ethnic’ or ‘country’ or ‘countries’ or ‘state?’ or ‘field?’ or ‘recovery’) 5 ‘post-conflict?’ or ‘postconflict?’ or ‘post-war?’ or ‘postwar?’ or (‘post’ next/2 (‘war?’ or ‘conflict?’ or ‘zone?’)) 6 ‘fragile’ next/2 (‘country’ or ‘countries’ or ‘region?’ or ‘state?’ or ‘setting?’)  7 ‘health’ next/2 (‘system?’ or ‘service?’) next/2 (‘reconstruction’ or ‘reconstructing’ or ‘rehabilitation’ or ‘rehabilitating’ or ‘rebuilding’) 8 #4 OR #5 OR #6 OR #7  9 #3 AND #8  10 #9 AND [2000-2021]/py |
| **SCOPUS (127)** | TITLE-ABS-KEY ("emergency medical service*" OR "emergency medical technician*" OR "emergency treatment*" OR "emergency medicine" OR "emergency service*" OR "emergency department*" OR "emergency room*" OR "emergency ward*" OR "emergency unit*" OR "emergency center*" OR "trauma center*" OR "emergency health service*" OR "accident and emergency" OR "accident & emergency" OR "a&e" OR "A & E" OR "ambulance*" OR "air ambulance*" OR "first aid” OR "prehospital" OR "pre-hospital" OR "paramedic" OR "out-of-hospital" OR "out of hospital" OR "ems" OR "emt" OR "emergency medical service" OR "emergency technician" OR "emergency practitioner" OR "emergency dispatch*" OR "first responder" OR "emergency rescue" OR "emergency resus" OR "emergency triage" OR "advanced life support" OR "emergency care practitioner" OR "prehospital" OR "pre-hospital" OR "emergency medical technicians" OR "emergency service*" OR "field triage" OR "out-of-hospital" OR "emergency medical service communication system*" OR "emergency medical dispatch*" OR "medical dispatch*" OR "telephone triage" OR "ambulance dispatch*" OR "Medical Priority Dispatch System")  AND  TITLE-ABS-KEY("post-conflict" OR "postconflict" OR "post-war" OR "postwar" OR "post-conflict area" OR "post-conflict zone" OR "post-conflict state" OR "post-conflict states" OR "post-conflict regions" OR "fragile state" OR "fragile states" OR "conflict recovery" OR "health system reconstruction" OR "health system rehabilitation" OR "health system reconstructing" OR "health system rehabilitating" OR "health system rebuilding" OR "health service reconstruction" OR "health service rehabilitation" OR "health service reconstructing" OR "health service rehabilitating" OR "health service rebuilding")  AND PUBYEAR AFT 2000 |
| **Web of Science (57)** | TS = (“emergency medical services” OR “emergency medical technicians” OR “emergency treatment” OR “emergency medicine” OR “ambulances” OR “air ambulances” OR “first aid” OR” military medicine” OR “prehospital” OR “pre-hospital” OR “paramedic” OR “ambulance” OR “out-of-hospital” OR “out of hospital” OR “ems” OR “emt” OR “emergency services” OR “emergency medical service” OR “emergency technician” OR “emergency practitioner” OR “emergency dispatch” OR “first responder” OR “public access defibrillation” OR “emergency rescue” OR “emergency resus” OR “emergency triage” OR “advanced life support” OR “emergency care practitioner” OR “extended care practitioner” ) OR TS=(“Ambulances” OR “Emergency Medical Technicians” OR “Air Ambulances” OR “emergency medical services” OR “paramedic” OR “ems” OR “emt” OR “prehospital” OR “pre-hospital” OR “first responder” OR “emergency medical technicians” OR “emergency services” OR “Ambulance” OR “field triage” OR “out-of-hospital”) OR TS=(“emergency medical service communication systems” OR “emergency medical dispatcher” OR “emergency medical dispatch” OR “medical dispatch” OR “telephone triage” OR “ambulance dispatch” OR “Medical Priority Dispatch System” )  AND  TS = (“post-conflict” OR “post conflict” OR “post-conflict setting” OR “postconflict setting” OR “post-conflict country” OR “postconflict country” OR “post-conflict countries” OR “post conflict countries” OR “post-conflict zone” OR “post conflict zone” OR “post-conflict region” OR “post conflict region” OR “post-war” OR “postwar” OR “post-war setting” OR “postwar setting” OR “post-war state” OR “postwar state” OR “post-war country” OR “postwar country” OR “post-war countries” OR “postwar countries” OR “post-war zone” OR “postwar zone” OR “post-war region” OR “postwar region”) OR TS= (“health system reconstruction” OR “health service reconstruction” OR “fragile setting” OR “fragile state” OR “fragile states” OR “fragile nation” OR “fragile nations” OR “fragile country” OR “fragile countries” OR “fragile region” OR “fragile regions” OR “conflict recovery”)   Date 2000-01-01 to 2021-09-09 |
| **Cochrane (3)** | MeSH: Emergency Medical Services Emergency Service, Hospital Emergency Medicine Keywords: "emergency medical service?" OR "emergency medical technician?" OR "emergency treatment" OR "emergency medicine" OR "ambulance?" OR "air ambulance?" OR "first aid” OR "prehospital" OR "pre-hospital" OR "paramedic?" OR "out-of-hospital" OR "out of hospital" OR "emergency service?" OR “emergency technician?" OR "emergency practitioner?" OR "emergency dispatch?" OR “first responder?” OR "public access defibrillation" OR "emergency rescue" OR "emergency resus" OR "emergency triage" OR "advanced life support" OR "emergency care practitioner?" OR "Emergency Medical Technician?" OR "Air Ambulances" OR "ems" OR "emt" OR "prehospital" OR "pre-hospital" OR "first responder?" OR "Ambulance" OR "HEMS" OR "field triage" OR "out-of-hospital" OR "emergency medical service communication system?" OR "medical dispatch?" OR "telephone triage" OR "ambulance dispatch?" OR "Medical Priority Dispatch System"  AND  MeSH: Armed conflicts OR  Keywords: "post-conflict?" OR "postconflict?" OR "post-war?" OR "postwar?" OR "post war?" OR "post conflict?" OR “post war zone?” OR “post-conflict area?” OR “post-conflict zone?” OR “post-conflict region?” OR “post-conflict state?” OR “post-conflict recovery” OR “ethnic conflict” OR “military conflict” OR “combat area?” OR “combat zone?” OR “combat region?” OR “fragile countr?” OR “fragile region?” OR “fragile state?” OR “fragile setting?” |
